# Genome Sequence of *Lactobacillus paracasei* Strain LC-Ikematsu, Isolated from a Pineapple in Okinawa, Japan

**DOI:** 10.1128/genomeA.01583-16

**Published:** 2017-02-02

**Authors:** Ipputa Tada, Seikoh Saitoh, Hiroaki Aoyama, Naoya Shinzato, Norikuni Yamamoto, Masanori Arita, Shinya Ikematsu

**Affiliations:** a Department of Genetics, SOKENDAI (Graduate University for Advanced Studies), Shizuoka, Japan; b Tropical Biosphere Research Center, University of the Ryukyus, Okinawa, Japan; c Center for Strategic Research Project, University of the Ryukyus, Okinawa, Japan; d Okinawa Ham Savoury Food Company, Okinawa, Japan; e Center of Information Biology, National Institute of Genetics, Shizuoka, Japan; f Department of Bioresources Engineering, National Institute of Technology, Okinawa College, Okinawa, Japan

## Abstract

The draft genome sequence of *Lactobacillus paracasei* strain LC-Ikematsu, isolated from a pineapple in Okinawa, was determined. The total length of the 87 contigs was 3.08 Mb with a G+C content of 46.2% and 2,946 coding sequences. The genome analysis revealed its biosynthetic ability of 11 amino acids.

## GENOME ANNOUNCEMENT

Lactic acid bacteria (LAB) are important industrial resources for food and silage industries (1). In our survey of subtropical *Lactobacillus* spp. of plant origin, one strain, *L. paracasei*, was found to reduce the cellular activity of tyrosinase, possibly by lipoteichoic acid (LTA), through a similar mechanism as in *L. plantarum* (2). Here, we report the draft genome sequence of *L. paracasei* strain LC-Ikematsu.

Two next-generation sequencers, Roche 454 GS Junior and Illumina MiSeq, were used for the sequencing. The genomic library was prepared with the GS rapid library preparation kit for Roche 454, and the Nextera DNA library preparation kit for Illumina MiSeq. A total of 88,722 single-end (500 bp) and 1,000,000 paired-end (150 bp) reads from each platform were used, corresponding to an 86-fold coverage in total. Then, both reads were combined and subjected to a *de novo* assembly with Newbler version 2.7 (Roche) with default parameters (3). The obtained draft genome consisted of 87 contigs (>500 bp), and the total size was 3,078,383 bp with an *N*_50_ value of 100.2 kb and a G+C content of 46.2%. The completeness (>99%) and contamination (0.5%) of the draft genome were assessed with CheckM software (4). Average nucleotide identity value was 98.4% between LC-Ikematsu and *L. paracasei* subsp. *paracasei* JCM 8130 (5).

The genome annotation was performed by DFAST, an annotation server specialized for *Lactobacillus* spp. (6). The KEGG Automatic Annotation Server (KAAS) was also used to identify metabolic pathways (7). Prophages and clustered regularly interspaced short palindromic repeat (CRISPR) loci were predicted with PHAST (8) and CRISPRFinder (9), respectively. The genome contained 2,946 protein-coding sequences, among which 2,235 were assigned functions. One copy of the 16S rRNA gene and one copy of the 23S rRNA gene were identified, but no 5S rRNA genes were identified, probably due to an assembly error. The total number of tRNAs was 48, similar to other strains (e.g., 61 for *L. paracasei* JCM8130, 58 for* L. casei* ATCC 393, and 56 for *L. rhamnosus* ATCC 53103 [10]). Six prophage regions were identified: three regions (40.5, 42.5, and 37.2 kb) were complete, two regions (46.9 and 15 kb) were partial, and one region (16.8 kb) was questionable. One CRISPR locus was detected, which contained four CRISPR-associated proteins and 26 spacer sequences separated by 36-bp direct repeats. The biosynthetic pathways for amino acids were well conserved in the LC-Ikematsu strain, and we could verify the requirement of nine amino acids (Glu, Leu, Ile, Tyr, Cys, Phe, Arg, Trp, and Val) on a synthetic culture medium. The predicted metabolic pathways are similar to previous reports (11).

Tyrosinase inhibition is a recently reported bioactivity in LAB (2). The variety and function of LTAs are diverse, and their role is also critical for probiotics (12). Investigation of the LTA type of the LC-Ikematsu strain will contribute to the understanding of the beneficial roles of LAB in general.

### Accession number(s).

This whole-genome sequencing project was deposited in DDBJ/ENA/GenBank under the accession number BDIT00000000. The version described in this paper is the first version, BDIT01000000.
